# Gut microbiomes of cycad-feeding insects tolerant to β-methylamino-L-alanine (BMAA) are rich in siderophore biosynthesis

**DOI:** 10.1038/s43705-023-00323-8

**Published:** 2023-11-22

**Authors:** Karina Gutiérrez-García, Melissa R. L. Whitaker, Edder D. Bustos-Díaz, Shayla Salzman, Hilda E. Ramos-Aboites, Zachary L. Reitz, Naomi E. Pierce, Angélica Cibrián-Jaramillo, Francisco Barona-Gómez

**Affiliations:** 1 Evolution of Metabolic Diversity Laboratory, Unidad de Genómica Avanzada (Langebio), Cinvestav-IPN, Km 9.6 Libramiento Irapuato - León, Irapuato, Guanajuato 36824 México; 2 Department of Embryology, Carnegie Institution for Science, 3520 San Martin Drive, Baltimore, MD 21218 USA; 3 Museum of Comparative Zoology, Department of Organismic and Evolutionary Biology, Harvard University, 26 Oxford Street, Cambridge, MA 02138 USA; 4 Department of Biological Sciences, East Tennessee State University, Johnson City, TN 37614 USA; 5 Institute of Biology, Leiden University, Sylviusweg 72, Leiden, 2333 BE The Netherlands; 6 University of Georgia, Entomology Department, Athens, GA 30602 USA; 7 Bioinformatics Group, Wageningen University, Droevendaalsesteeg 1, 6708PB Wageningen, The Netherlands; 8 Ecological and Evolutionary Genomics Laboratory, Unidad de Genómica Avanzada (Langebio), Cinvestav-IPN, Km 9.6 Libramiento Irapuato - León, Irapuato, Guanajuato 36824 México; 9 Naturalis Biodiversity Center, Darwinweg 2, 2333 CR Leiden, The Netherlands

**Keywords:** Microbiome, Microbial ecology

## Abstract

Ingestion of the cycad toxins β-methylamino-L-alanine (BMAA) and azoxyglycosides is harmful to diverse organisms. However, some insects are specialized to feed on toxin-rich cycads with apparent immunity. Some cycad-feeding insects possess a common set of gut bacteria, which might play a role in detoxifying cycad toxins. Here, we investigated the composition of gut microbiota from a worldwide sample of cycadivorous insects and characterized the biosynthetic potential of selected bacteria. Cycadivorous insects shared a core gut microbiome consisting of six bacterial taxa, mainly belonging to the Proteobacteria, which we were able to isolate. To further investigate selected taxa from diverging lineages, we performed shotgun metagenomic sequencing of co-cultured bacterial sub-communities. We characterized the biosynthetic potential of four bacteria from *Serratia*, *Pantoea*, and two different *Stenotrophomonas* lineages, and discovered a suite of biosynthetic gene clusters notably rich in siderophores. Siderophore semi-untargeted metabolomics revealed a broad range of chemically related yet diverse iron-chelating metabolites, including desferrioxamine B, suggesting the occurrence of an unprecedented desferrioxamine-like biosynthetic pathway that remains to be identified. These results provide a foundation for future investigations into how cycadivorous insects tolerate diets rich in azoxyglycosides, BMAA, and other cycad toxins, including a possible role for bacterial siderophores.

## Introduction

Cycads are among the oldest seed plants, with a lineage that traces back to the Permian [1]. The genome of one cycad species in the genus *Cycas* was recently published, and with more than 10 Gbp, suggests a series of gene expansions as well as a whole genome duplication prior to divergence from its gingko sister taxa [2]. These tropical gymnosperms harbor defensive chemicals in their leaves and other tissues, including the azoxyglycoside methylazoxymethanol (MAM), or cycasin, and the non-proteinogenic amino acid β-Methylamino-L-Alanine (BMAA), both of which are toxic to diverse organisms [3, 4]. Previous studies have demonstrated that several insect herbivores are able to detoxify and even sequester the cycad-derived MAM glycoside, cycasin [4–7], whereas mechanisms of BMAA resistance, just as its biosynthesis, remain elusive [8, 9]. BMAA is capable of blocking glutamate receptors [3], causing neurotoxic damage in insects [10, 11] and mammals [12] as well as developmental problems in *Arabidopsis thaliana* [13]. This contrasts with the recent observation that BMAA quantities in cycad leaves may not be acutely toxic or deterrent to insects [14].

In addition to MAM and BMAA, the genomes of a few species of *Cycas* cycads encode for a functional FitD insect toxin that was acquired by horizontal gene transfer from a microbial source, and then expanded into multiple copies [2]. Many bacteria also have *fitD* genes, as well as the homologous genes *mcf* (‘makes caterpillar floppy’), which have been shown to be broadly distributed and to contribute to insecticidal activity in plant- and nematode-associated bacteria [15, 16]. These observations, together with BMAA and MAM toxicity, suggest that cycads’ diverse specialized metabolites exert significant evolutionary pressure on cycad-specialized insects and other organisms. Even so, several specialized insect species are able to feed on cycad tissues without adverse effects, including some beetles, thrips, moths, and butterflies [17, 18], consistent with the so-called “gut microbial facilitation hypothesis” [19]. At least two species of cycad-feeing Lepidoptera sequester BMAA in their larval and adult tissues [18], and for at least one group of cycad specialists, *Eumaeus* butterflies, toxin tolerance appears to be a key innovation in the evolution of these insects [20].

The gut bacterial communities of many insects are essential for nutrient acquisition, digestion, and detoxification [21]. Many microorganisms are capable of enzymatically degrading plant-specialized metabolites, and growing evidence suggests that symbiotic gut bacteria can help herbivorous insects cope with diets rich in plant defensive chemicals [22], and can play a role in resistance to insecticides [23, 24]. However, despite the overall effect of plant secondary metabolites on the plant, insect, and soil microbiomes, which have been broadly documented [25, 26], examples of phytochemicals specifically affecting the metabolomes of these microbiomes are scarce or nonexistent [27, 28]. An earlier study of the microbiota of cycadivorous insects identified a small core of bacterial taxa that are shared across phylogenetically and geographically distinctive insects [29]. The present study aimed to analyze the gut bacteria of a larger sampling of cycad feeding insects, and to explore possible functions by assessing specialized metabolites biosynthesized by sub-communities and selected bacterial taxa isolated from the guts of these insects.

To do so, we characterized the bacterial communities of cycadivorous insects from around the world using 16 S sequencing, and then applied *EcoMining* [30], a method of capturing bacterial groups and metabolites of interest by co-culturing bacterial sub-communities for shotgun metagenomics, phylogenomics, and integrated omics analyses. The advantage of this method is that it allows for the distillation of functionally relevant fractions of the entire microbiome based on a priori biological hypotheses. We then investigated the metabolic functions of selected bacterial taxa by mining for biosynthetic gene clusters (BGCs) predicted to direct the synthesis of specialized metabolites, and in some cases, confirming the expression of diverse yet related siderophores through semi-untargeted siderophore metabolomics. Our results provide insight into the functional capabilities of bacteria found in the guts of cycadivorous insects, and their potential importance for chemically-mediated cycad-insect interactions.

## Materials and methods

### Microbiome global sampling of cycadivorous insects

**Fig. 1:**
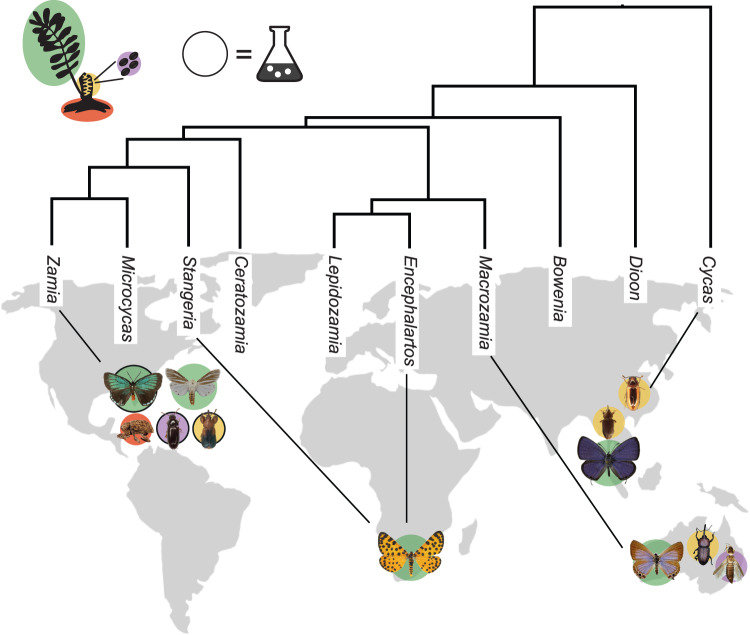
Cycadivorous insect sampling strategy. Coleoptera, Lepidoptera, and Thysanoptera were collected from four continents. Black lines connect each insect species to the cycad genus on which it feeds, and background colors represent the plant tissue on which each insect is specialized: green = leaves, yellow = cones, purple = pollen, and red = roots. All cycad herbivores encounter cycad toxins, regardless of the plant tissue on which they feed. Dark black outlines indicate the three insect genera that were subjected to the *EcoMining* approach.

With the aim of capturing as much ecological and trophic diversity as possible, 12 insect species from 3 orders were collected from the USA, South Africa, China, Australia, Singapore, and Thailand between 2014 and 2017 (Fig. 1). Over 90 insect samples were included in the analysis, with detailed collecting information in Table S1. All species included in the study are obligate cycad specialists with two exceptions: the neotropical moth, *Seirarctia echo* (Erebidae), which feeds on cycads and plants from multiple families of angiosperms [17], although the specimens we analyzed were collected from *Zamia* cycads; and the larvae of the African moth *Zerenopsis lepida*, which are obligate cycad folivores in early instars, although some individuals switch to feeding on angiosperms in the fourth instar [31]. We were able to collect late instar *Z. lepida* feeding on *Encephalartos* and *Stangeria* cycads as well as an angiosperm (*Maesa lanceolata*).

Insects were processed differently depending on collector, insect type, and size. Lepidopteran larvae in their third instar or older were collected with sterile forceps, submerged in ethanol, and rinsed in sterile PBS. Their guts were then excised using flame-sterilized dissecting tools. Dissected guts were preserved in individual vials containing 97% ethanol. The guts of *Zerenopsis lepida* were not dissected due to their small size; instead, they were surface-sterilized using a 10% bleach solution and preserved whole. Coleopteran specimens were either immediately flash frozen in liquid nitrogen or preserved in ethanol. Whole specimens were then submerged for 5 s in a 10% bleach solution and rinsed in PBS for surface sterilization prior to DNA extraction. Finally, specimens from the thysanopteran species *Cycadothrips chadwicki* were neither surface sterilized nor dissected due to their small size; instead, approximately 150 individual thrips were pooled together into a single sample for DNA extraction.

### Amplicon metagenomics of the cycadivorous insect’s gut microbiome

DNA extractions were performed using the Powersoil DNA isolation kit (MoBio Laboratories, Carlsbad, CA, USA) according to the manufacturer’s instructions with the addition of 60 μg of proteinase K to the lysis buffer prior to bead-beating. DNA concentration was assessed using a Qubit Fluorometer (Invitrogen, Carlsbad, CA, USA), and samples with low DNA yields were concentrated as per the DNA extraction kit’s recommendations. DNA extracts were sent to Argonne National Laboratories (Lemont, IL, USA) for library preparation and 16 S rRNA gene amplification and sequencing. Amplification of the 16 S rRNA was done using barcoded primers 515 F (5′-GTGYCAGCMGCCGCGGTAA-3′) and 806 R (5′-GGACTACNVGGGTWTCTAAT-3′) of the V4 region [32], and libraries were pooled and sequenced with Illumina MiSeq instrument in 2 × 150 paired-end mode. Raw sequences were processed following previously published methods [33]. Briefly, sequences were demultiplexed and quality filtered in QIIME [34] then de novo clustered at 97% identity using UPARSE [35]. QIIME was used to assign taxonomy and to construct a phylogenetic tree of representative sequences. The resulting BIOM table and phylogenetic tree were exported to R and analyzed with the phyloseq [36], the phyloseq-extended [37], and the microbiome packages. Non-target sequences (mitochondria, chloroplasts, *Wolbachia*, and the common lab contaminants *Escherichia* and *Staphylococcus*) were removed from the dataset and spurious sequences were identified and removed using the PERFect package’s permutation filtering method implemented with the “fast” algorithm [38]. We defined a species’ core microbiome as the set of bacterial operational taxonomic units (OTUs) present in at least 83% of replicates within a species (corresponding to 5 out of 6 replicates, for example). For species represented by multiple life stages (e.g., larva & adult) or, in the case of *Zerenopsis lepida*, multiple host plants, cores were calculated separately for each grouping. For samples with only a single replicate (e.g., the pooled *Cycadothrips* sample), a within-species core could not be obtained, but these samples were included in an across-species core, defined as OTUs present in 98% of all samples.

### Co-culture inoculation for shotgun metagenomics and bacterial isolation

Three insect species were collected and processed to provide inocula for co-cultures: *Rhopalotria slossoni* (Coleoptera), *Pharaxanotha floridana* (Coleoptera), and *Eumaeus atala* (Lepidoptera) (Table S1). Twenty-two *R. slossoni* adults, fourteen *P. floridana* adults, and two *E. atala* larvae were surface sterilized by successive washes in 70% ethanol and sterile dd-MilliQ water. The guts of *E. atala* larvae were excised using sterile dissecting tools and then suspended, individually, in sterilized dd-MilliQ water and homogenized with a sterile micropestle. Coleopteran specimens were pooled by species, suspended in sterilized dd-MilliQ water, and homogenized with a sterile micropestle. Resulting biomass from each sample was divided to inoculate two 100 ml co-cultures per sample of liquid Brain Heart Infusion (BHI) medium (Becton Dickinson, Germany) at 0.05x, one without BMAA and one with 20 µM of BMAA, a concentration used in previous studies showing the toxic effects on insects [39, 40]. Co-cultures were incubated at 23 °C with agitation at 150 r.p.m for 48 h, after which each co-culture was used to inoculate petri dishes containing different bacterial media described below. DNA was extracted from the eight co-cultures for shotgun metagenomics. Two additional co-cultures (BMAA +/-) were generated from the pooled *P. floridana* biomass and were used for genomic isolation but not shotgun metagenomics.

### Shotgun metagenome sequencing and taxonomic analysis

DNA was extracted from co-cultures using a standard phenol-chloroform protocol [41], and DNA quality was checked by Nanodrop 2000/2000c (Thermo Scientific, USA). Libraries were prepared with the Truseq nano kit and sequenced at UGA, Cinvestav (Irapuato, Mexico) using the NextSeq Illumina platform 2 × 150 paired-end reads format. Obtained reads were checked with FastQC v0.11.9 [42], and low-quality bases were trimmed with Trimmomatic v0.32 [43]. Metagenomic profiling of high-quality paired reads was performed using kraken2 [44] with the maxikraken database (September 2018 release; available at https://lomanlab.github.io/mockcommunity/mc_databases.html). Abundances, taxonomic lineage tables, and metadata were exported to R using the kraken-biom software [45] to be analyzed with the phyloseq [36], the taxize [46], and the microbiome packages.

### Shotgun metagenome assembly, mining, and binning

Assembly of the six metagenomes obtained was performed with metaSPAdes v3.10 using default parameters [47]. The resulting scaffolds were annotated using RAST [48], taxonomically classified using kraken2 with the same database and parameters used for read classification, and mined using antiSMASH v5.0 [49], BiG-SCAPE and CORASON [50]. To obtain the core biosynthetic gene clusters (BGCs), each complete and non-redundant BGC obtained from antiSMASH was used to construct a presence/absence matrix. A core BGCs plot was then created using the UpSetR R package v1.4.0 [51], with all the set intersections in this matrix. Scaffolds were binned with the PATRIC Metagenomic Binning Service [52] using default parameters, from which nine metagenome-assembled genomes (MAGs) were obtained: 4 for *Stenotrophomonas*, 4 for *Serratia*, and 1 for *Pantoea* (Table S2). To obtain 16 S sequences from the metagenomes we used the 16 S sequence recovery pipeline of Anvi’o [53].

### Phylogenomic analysis of selected bacteria

To establish the relationships between MAGs and genome sequences of bacterial isolates, phylogenies of *Stenotrophomonas*, *Pantoea*, and *Serratia* were constructed using representative genomes from each genus, along with our newly generated genome sequences and recovered MAGs (Tables S3, S4, and S5). For each phylogeny, a protein core was obtained using Get-Phylomarkers [54], computed with Get-Homologues using the algorithms bidirectional best-hit (BDBH), Clusters of Orthologous Groups-triangles (COGtriangles), and OrthoMCL (Markov Clustering of orthologs, OMCL) with default parameters (Tables S6, S7, and S8). Resulting matrices, containing 39, 64, and 712 proteins, respectively, were used to construct phylogenetic trees with MrBayes v3.2 [55] with a mixed substitution model based on posterior probabilities (aamodel[Wag]1.000) for proteins for 100,000 generations. The trees were visualized with FigTree v1.4.2 [56].

### Isolation and identification of bacteria from co-cultures in semi-selective media

Semi-selective media were chosen to target specific bacterial phyla that are known to be represented in cycadivorous insects’ microbiomes [29]. These were: 1) SFM media, (mannitol: 20 g/L; soya flour 20 g/L; and agar 17 g/L for solid media); 2) ISP4 and ISP4N-, (starch: 10.0 g/L; dipotassium phosphate: 1 g/L; magnesium sulfate: 1 g/L; sodium chloride: 1 g/L; ammonium sulfate: 2 g/L for ISP4 media, none for ISP4N- media; calcium carbonate: 2 g/L; ferrous sulfate: 1 mg/L; magnesium chloride: 1 mg/L; zinc sulfate: 1 mg/L; final pH 7.0; and agar 17 g/L for solid media); 3) BHI media at concentrations 0.02x and 0.05x (Becton Dickinson, Germany); 4) FLA media (Fortified lipid agar) (tryptic soy broth: 16 g/L; vegetable oil: 10 mL/L; nutrient broth 12 g/L; yeast extract: 5 g/L; and agar 17 g/L for solid media); and 5) NTBA media (peptone: 5 g/L; beef extract: 3 g/L; bromothymol blue: 0.025 g/L; 2,3,5-triphenyl tetrazolium chloride: 0.04 g/L; and agar 17 g/L for solid media).

The Petri dishes were incubated at 22 °C for 72 h. After this, only unique bacterial morphotypes were selected for further isolation and purification in their corresponding media. Once bacterial morphology was homogeneous on each isolation plate, isolates were grown on 100 ml of their corresponding media for 48 h. DNA extraction and quality measurements were done as before. gDNA was used for PCR amplification of the 16 S rRNA region using the universal bacterial primers 27 F and 1492 R [57]. The amplification yielded PCR fragments of 1.4 Kbp in length, which were purified with the Qiagen QIAquick PCR purification kit (Hilden, Germany) and sequenced using Sanger chemistry at UGA, Cinvestav (Irapuato, Mexico). Taxonomic identity of the sequences was determined using Blastn against the SILVA rRNA database v132 [58]. Each sequence was classified according to the best hit (>97% sequence identity) (Table S9). An alignment of all of the obtained 16 S rRNA sequences was constructed with ClustalW [59] and edited with Gblocks v0.91 to remove ambiguous positions [60]. A phylogenetic tree was constructed using MrBayes v3.2 [55], with a 4by4 nucleotide substitution model for 100,000 generations, with sampling every 100 generations. The graphical representation of the phylogenetic trees was obtained with Iroki Tree Viewer [61].

### Genomic sequencing and natural products mining of isolated strains

We selected six strains of interest from the isolates based on two criteria: 1) their prevalence in the gut microbiomes of *R. slossoni*, *P. floridana*, and *E. atala*; and 2) their biosynthetic potential to produce conserved natural products. For genomic characterization of these strains, we grew monocultures of the selected *Serratia, Pantoea*, and *Stenotrophomonas* strains, and the obtained biomass was used for DNA extraction and quality measurements as before. Libraries were prepared using the Truseq nano kit and sequenced at UGA, Cinvestav (Irapuato, Mexico) using the NextSeq Illumina platform with 2 × 150 paired-end reads format. Raw reads were checked with FastQC v0.11.9 and low-quality bases were trimmed using Trimmomatic v0.32 [43]. Multiple de novo genomes were assembled with SPAdes v3.15.3 using default parameters [62] and annotated with RAST [48] (Table S2). CORASON [50] was used to mine turnerbactin-like siderophores in the different MAGs and genomes within a phylogenetic context. CORASON hits that contained homologs of the turnerbactin-like non-ribosomal peptide synthetase (NRPS) were obtained using a BlastP (*e*-value and cut-off of 0.001).

### Siderophore genome mining

To further characterize the putative siderophore BGCs detected by antiSMASH v5.0 [49], we functionally annotated genes in the BGCs that were previously implicated in iron metabolism. This included enzymes annotated as acyl CoA-dependent acyltransferases, FAD-dependent amine monooxygenases, and iron reductases, as well as TonB-dependent receptors and ABC transporters. The presence of conserved DNA motifs corresponding to iron boxes, known to be involved in iron regulation in bacteria [63], was also determined and used to mine the genomes. An iron box database was constructed using DNA motifs previously described for Gram-positive bacteria (TTAGGTTAGGCTCACCTAA and TGATAATNATTATCA) [64, 65], and Gram-negative bacteria (GATAATGATAATCATTATC, GATAAAATTAATCAGCCTC, and ATTAATAAAAACCATTGTC, GATAATGAGAATCATTATT, GATAATTGTTATCGTTTGC, TATAATGATACGCATTATC, TGTAATGATAACCATTCTC, GAATATGATTATCATTTTC, GAAAATGATAATCATATTC, ATAAATGATAATCATTATT, GATAATCATTTTCAATATC) [64, 66, 67]. The presence of iron box motifs was analyzed with PREDetector v3.1 [68] using the iron box motifs dataset.

### Identification of bacterial siderophores through HPLC and MS

The *Pantoea*, *Serratia*, and *Stenotrophomonas* bacteria were subjected to semi-untargeted siderophore extraction and characterization. Each strain was cultivated in two separate Erlenmeyer flasks containing LB medium, with and without 200 µM of the iron-chelator compound 2,2′-dipyridyl (DIPY) for four days, as previously [65]. Culture growth was checked by OD_600_ measurements every 12 h and cultures were harvested upon reaching saturation. Cultures were then centrifuged at 8000 rpm and the supernatant was collected and lyophilized. Dry extracts were resuspended in 10 ml of sterile dd-MilliQ water and separated in two Falcon tubes, each with 5 ml of the resuspended extracts. Iron-chelating metabolites were converted to their ferric complexes by addition of 1 M FeCl_3_ to one of the tubes of each condition. All samples were then concentrated by filtering the supernatants with Millipore Millex-GN filters with a nylon membrane of 0.22 µm. Samples were analyzed on a Thermo Ultimate 3000-uHPLC instrument equipped with a quaternary pump, a diode array detector, and a ZORBAX Eclipse XDB-C18 (Agilent, USA) analytical column (4.6 × 150 mm, 5 µm). Analytical conditions were as follows: The mobile phase comprised a binary system of eluent A, 0.1% trifluoroacetic acid, and eluent B, 100% acetonitrile. The run consisted of a gradient from 0 to 100% B for 25 min. Tris-hydroxamate-Fe^3+^ complexes, formed after the addition of iron chloride to bacterial extracts, were detected at a wavelength of 435 nm. Fractions were collected and pooled by bacterial genus for mass spectrometry (MS) analysis in an ion trap LTQ Velos (Thermo Scientific, Waltham, USA). MS/MS analysis of selected ions was performed with a collision energy of 20 eV, and spectra were analyzed using the XCalibur software v3.0 (Thermo Scientific, USA). Metal-chelating metabolites were identified by comparing fragmentation patterns with those of siderophores previously reported [65] or included in the Bertrand’s siderophore database (http://bertrandsamuel.free.fr/siderophore_base/index.php) and the GNPS database used to generate GNPS feature-based molecular networks [69, 70].

## Results

### 16 S taxonomic profiling of cycadivorous insects’ guts identifies a global microbiome core

**Fig. 2:**
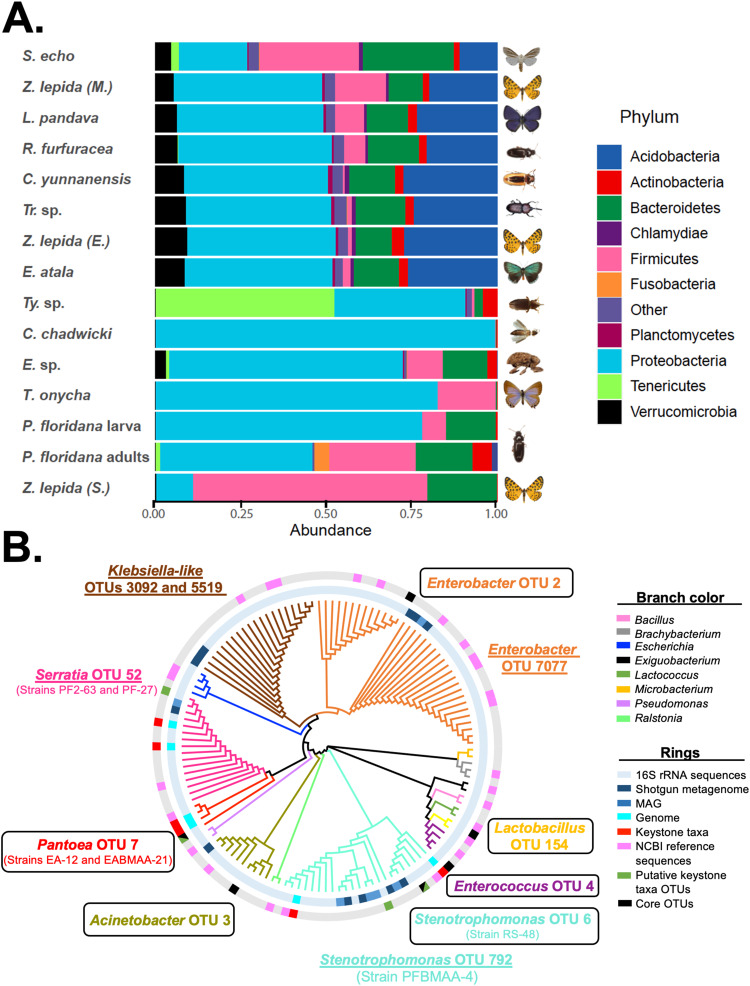
Composition and distribution of cycadivorous insect’s gut microbiome. **A** Community composition among a global sampling of cycad-feeding insects. Proteobacteria dominate the gut microbiomes of most cycadivorous insects, followed by Firmicutes and Bacteriodetes. **B** 16 S rRNA phylogenetic tree including 79 bacterial strains isolated from the guts of *Eumaeus atala*, *Pharaxanotha floridana*, and *Rhopalotria slossoni*. The 16 S rRNA gene from the six genomes of the genera *Serratia, Pantoea*, and *Stenotrophomas*, plus all the metagenomes data and the 16 S amplicon data were included. Core taxa identified after the integration of different datasets are highlighted: core taxa are shown within boxes and semi-conserved taxa shown underlined. Specific strains selected for further experimental characterization are shown between parentheses below their cognate OTUs.

16 S amplicon sequencing of DNA extracted from the guts of a global sampling of cycadivorous insect species yielded 6,317,577 bacterial reads. After removing non-target and spurious OTUs, we were left with 5,027,153 reads across 92 samples. Observed bacterial species richness ranged from 41 to >700 OTUs within a single insect sample. A total of 26 bacterial phyla were detected (Fig. 2A), with Proteobacteria comprising moderate to dominant portions of the microbiomes of most insects with a few exceptions: *Zerenopsis lepida* larvae feeding on *Stangeria* cycads (but not other plants) were dominated by Firmicutes, *Tychiodes* sp. adults were dominated by Tenericutes, and *Seirarctia echo* larval microbiomes were composed of relatively equal proportions of Proteobacteria, Bacteroidetes, and Firmicutes. Insects’ core microbiomes ranged widely in richness, from 17 core OTUs in *Luthrodes pandava* larvae (*n* = 7) to 185 OTUs in *Eubulus* sp. larvae (*n* = 7). In general, the core microbiomes of Coleoptera tended to be larger than in Lepidoptera. A cross-species core included 6 OTUs found in all insect species, with highly variable relative abundances across samples. Some core OTUs differ slightly from previously reported core bacteria [29], likely due to methodological differences in how the core was defined between studies. In the present study, core OTUs belong to the genera *Acinetobacter* (OTU 3), *Pantoea* (OTU 7), *Enterobacter* (OTU 2), *Enterococcus* (OTU 4), *Lactobacillus* (OTU 154), and *Stenotrophomonas* (OTU 6). These taxa were then placed within a phylogenetic context (Fig. 2B) following isolation and analysis of selected bacterial species, as described in the following sections.

### Isolation of bacteria from the microbiomes of cycadivorous insects’ guts

To direct bacterial isolation towards ecologically relevant strains from the broad diversity of cycadivorous insects investigated, we adopted the *EcoMining* approach, which we have successfully employed to investigate the microbiome of cycad’s coralloid roots and its biosynthetic potential [71]. We obtained biacterial isolates from the sub-community co-cultures of the dissected guts of *Eumaeus atala, Pharaxanotha floridana*, and *Rhopalotria slossoni* (Fig. 1, circles). This approach led to two types of cultures for further analyses: (i) bacterial isolates obtained in selective and semi-selective media, which were characterized after amplification and sequencing of their 16 S rRNA gene, and eventually their whole genome for selected strains; and (ii) sub-community co-cultures grown under functionally relevant conditions, such as the presence of the cycad toxin BMAA, which were shotgun sequenced to identify core taxonomic and functional genes, both at the single-gene level and as part of metagenome-assembled genomes (MAGs) (Table 1).

**Table 1:** Metagenomes and genomes generated in this study.

(Meta)genome sequence/strain	Type	Bacterial genus	Origin^a^	Accession number^b^
EA	Metagenome	NA	EA	SRR24296969
EABMAA	Metagenome	NA	EA + BMAA	SRR24296968
PF	Metagenome	NA	PF	SRR24296967
PFBMAA	Metagenome	NA	PF + BMAA	SRR24296966
RS	Metagenome	NA	RS	SRR24296965
RSBMAA	Metagenome	NA	RS + BMAA	SRR24296964
Pa-EAmG	MAG	*Pantoea*	EA	SAMN34374683
Se-PFmG	MAG	*Serratia*	PF	SAMN34374684
St-PFmG	MAG	*Stenotrophomonas*	PFBMAA	SAMN34374685
Se-PFBMAAmG	MAG	*Serratia*	PFBMAA	SAMN34374686
St-PFBMAAmG	MAG	*Stenotrophomonas*	PFBMAA	SAMN34374687
Se-RSmG	MAG	*Serratia*	RS	SAMN34374688
St-RSmG	MAG	*Stenotrophomonas*	RS	SAMN34374689
Se-RSBMAAmG	MAG	*Serratia*	RSBMAA	SAMN34374690
St-RSBMAAmG	MAG	*Stenotrophomonas*	RSBMAA	SAMN34374691
EA-12	Genome	*Pantoea*	EA	SAMN34373067
EABMAA-21	Genome	*Pantoea*	EABMAA	SAMN34373068
PFBMAA-4	Genome	*Stenotrophomonas*	PFBMAA	SAMN34373069
RS-48	Genome	*Stenotrophomonas*	RS	SAMN34373070
PF2–63	Genome	*Serratia*	PF2	SAMN34373071
PF-27	Genome	*Serratia*	PF	SAMN34373072

*NA* not applicable, *EA* *Eumaeus atala*, *PF* *Pharaxanotha floridana*, *RS* *Rhopalotria slossoni*.

^a^The origin refers to the co-culture from which the metagenome DNA was extracted and sequenced or the bacterial strain isolated and subsequently sequenced.

^b^NCBI accession number.

To compare the metagenomics datasets generated from cultures (i.e., EcoMining) and culture-independent methods (16 S profiling), we extracted a subset of the 16 S profiling data from *Rhopalotria furfuracea* adults (*n* = 6), *Pharaxanotha floridana* adults (*n* = 7), and *Eumaeus atala* larvae (*n* = 5), for comparison to the taxonomic information generated by co-culture shotgun metagenomic sequencing. Alpha diversity analysis showed that shotgun metagenomes recovered more OTUs than 16 S amplicon sequencing (Fig. S1A) and that these OTUs belonged to fewer phyla, with Proteobacteria being the most dominant phylum, reflecting a partial but marginal growth bias of the co-cultures (Fig. S1B). These analyses also showed that addition of BMAA to the co-cultures does not alter the taxonomic composition of bacterial communities; instead, host species identity was the biggest factor contributing to taxonomic dissimilarities among sub-communities (Fig. S1A). The fact that BMAA did not alter the microbial diversity of the co-cultures is congruent with the fact that the isolated sub-communities are adapted to this toxin present in the insect’s diet.

Having assessed the validity of the co-culture metagenomes to recover core taxa identified by the 16 S metagenomic profiling, we sought to identify bacteria present in all of the sub-community co-cultures. We found that the Proteobacteria diversity recovered from the shotgun metagenomes and their corresponding 16 S sequences included the genera *Serratia, Acinetobacter, Stenotrophomonas*, and *Enterobacter* across all three insect species, albeit with different patterns of prevalence (Fig. S2). Given that Proteobacteria was also the most abundant phylum in the 16 S amplicon dataset, it is reasonable to conclude that this is a constitutive part of the insects’ gut microbiomes. 16 S metagenomic profiling identified several bacteria from the Enterobacteriaceae family that were not found in cultures but these taxa were only present in the metagenomes obtained from *Pharaxanotha floridana* with and without BMAA (PF and PFBMAA, respectively), and *Rhopalotria slossoni* (RS). Based on the 16 S phylogeny (Fig. 2B), the closest match to these previously uncultured bacteria belongs to the genus *Klebsiella* (OTUs 3092 and 5519), with sequence identity scores between 90% and 93% depending on the metagenome.

Using this integrated dataset as a guide, we then selected for further experimental characterization the following strains that we could isolate and confirm by direct sequencing of their rRNA 16 S gene: (i) EA-12 and EABMAA-21 as representatives of the *Pantoea* ‘OTU 7’ from *E. atala*; (ii) strains PF2–63 and PF-27 as representatives of the *Serratia* ‘OTU 52’ from *P. floridana*; and (iii) the strains PFBMAA-4 and RS-48, as representative of the *Stenotrophomonas* ‘OTU 792’ and ‘OTU 6’ from *P. floridana* and *R. slossoni*, respectively. Interestingly, these latter strains represent two distinct lineages within the *Stenotrophomonas* genus. These OTUs are either present or ubiquitous in the microbiomes of all insect species from the global sampling (Fig. S2, Table S10). We also identified and isolated strains of the previously mentioned uncultured core *Klebsiella*-like genus, but further analysis of these isolates was not pursued due to our inability to obtain representative MAGs and the uncertainty about their taxonomic classification needed for phylogenomics analyses.

### Phylogenomics of natural products highlights bacterial siderophore BGCs

**Fig. 3:**
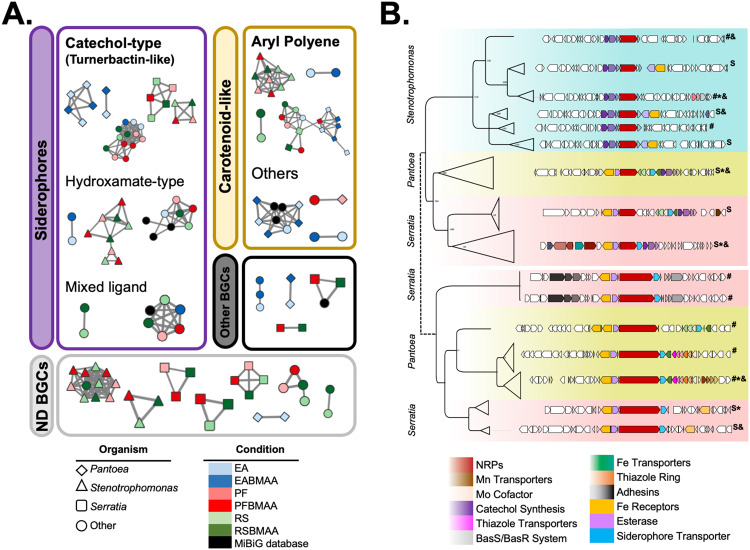
Conserved BGCs of cycadivorous core gut microbiome. **A** BiG-SCAPE networks containing 85 complete and non-redundant BGCs identified in the six metagenomes obtained from the *EcoMining* experiments. Turnerbactin-like, catechol-type siderophore, and aryl polyene BGCs formed five and four clans, respectively. **B** CORASON analysis with 230 turnerbactin-like BGCs from *Serratia, Pantoea*, and *Stenotrophomonas* genomes were used to reconstruct a BGC phylogeny. Turnerbactin-like BGCs can be separated into two groups based on changes in the NRPs’ protein sequences and their genomic context. The first group was found in all three bacterial genera, while the second was found only in *Serratia* and *Pantoea*. Two types of divergently related BGCs were found: (i) *bona fide* catechol-type siderophore BGCs (marked with S), and (ii) siderophore-like BGCs, no Fur box detected (#). BGCs found in the isolated strains from co-cultures are denoted with an asterisk (*), and BGCs found in MAGs with ampersands (&). EA, *Eumaeus atala* (BMAA + /-); PF, *Pharaxonotha floridana* (BMAA + /-); RS, *Rhopalotria slossoni* (BMAA +/-). Expanded versions of the trees supporting this general phylogeny are provided as supplementary information (Figs. S8, S9, S10, and S11).

Metagenomes from the six sub-community co-cultures were analyzed with antiSMASH and found to contain a total of 262 predicted BGCs, of which 85 were complete and non-redundant. These included 16 different types of BGCs, the most abundant of which were biosynthetic systems for highly conserved aryl polyenes and divergent non-ribosomal peptides (NRPs), with 20 and 19 BGCs, respectively (20% of the total). The remaining BGCs were bacteriocins (8), terpenes (11), NRPS-independent siderophores (5), hybrid PKS-NRPs (3), thiopeptides (3), resorcinols (2), butyrolactone (2), Hserlactone (1), type III PKS (1), lasso peptide (1), acyl amino acids (1), type II PKS (1), ectoine (1), and others (6) (Table S11). Of these, two core BGCs were present in all *EcoMining* metagenomes from the three insects: one turnerbactin-like divergent BGC from the catechol-type siderophore category, and one highly conserved carotenoid-like aryl polyene (Fig. S3, Table S12). Further analysis of these two core BGCs using BiG-SCAPE revealed that the turnerbactin-like and aryl polyene BGCs formed five and four clans, respectively, mostly composed of scaffolds assigned to *Pantoea* “OTU 7” mainly from the *E. atala* metagenomes, and *Serratia “*OTU 52” and *Stenotrophomonas* “OTU 6” and “OTU 792” mainly from the *R. slossoni* metagenomes. While the aryl polyenes are highly conserved the siderophores BGCs showed signs of diversification. For instance, other siderophore BGCs of the hydroxamate and mixed ligands types were found in *Stenotrophomonas* and other bacterial genera, such as *Acinetobacter*, *Klebsiella*, and *Enterobacter* (Fig. 3A).

To explore the evolutionary dynamics of the biosynthetic potential of these bacterial taxa, whole BGC CORASON phylogenies and core proteome species phylogenies were obtained. The *Pantoea* phylogeny indicates that *Pantoea* sp. EA-12 and *Pantoea* sp. EABMAA-21 are part of a monophyletic clade that includes *Pantoea* species previously isolated from plants and rivers, while *Pantoea* sp. Pa-EAmG belongs to a monophyletic clade of *Pantoea* isolated from insects, soil, and plants (Fig. S4). In the *Serratia* phylogeny, *Serratia* sp. PF2–63 and *Serratia* sp. PF-27 formed a monophyletic clade with MAGs *Serratia* sp. Se-RSBMAAmG, *Serratia* sp. Se-PFmG, *Serratia* sp. Se-PFBMAAmG, and *Serratia* sp. Se-RSmG, and *Serratia* sp. OMLW3, an isolate from the insect, *Orius majusculus* [72] (Fig. S5). The *Stenotrophomonas* phylogeny was composed of two monophyletic clades, one containing the genomes and MAGs from *Stenotrophomonas* sp. RS-48, *Stenotrophomonas* sp. St-RSmG, and *Stenotrophomonas* sp. St-RSBMAAmG, plus isolates from soils and rivers; and the other composed of *Stenotrophomonas* sp. PFBMAA-4, *Stenotrophomonas* sp. St-PFmG, and *Stenotrophomonas* sp. St-PFBMAAmG (Fig. S6). These phylogenies place the strains within an evolutionary context specific to the insect’s gut, and provide a framework to look into BGC distribution.

A contrasting evolutionary dynamic was found for these two conserved BGCs. On one hand, the carotenoid-like aryl polyene BGC was highly conserved across the phylogenies of the core bacteria *Pantoea* “OTU 7”, *Serratia* “OTU 52”, and *Stenotrophomonas* “OTU 6 and OTU 792”, with changes limited to taxonomic distance (Fig. S7). In contrast, the turnerbactin-like BGCs, for which in-depth functional annotation of each locus was performed, were composed of two types of divergently related BGCs: (i) *bona fide* catechol-type siderophore BGCs and (ii) siderophore-like BGCs for which some of the expected key functional elements for a siderophore BGC, e.g., regulatory iron boxes could not be detected (Figs. S8, S9, S10, and S11). This analysis also showed that turnerbactin-like BGCs could be separated into two groups based on changes in the NRP protein sequences and their genomic vicinity. The first group was found in all three bacterial genera, while the second was found only in *Serratia* and *Pantoea* (Fig. 3B). The gene composition of the BGCs—i.e., the presence or absence of genes for recognition of certain metals such as Fe, Mn, and Mo—further separated the turnerbactin-like BGCs found in each genus.

### Semi-untargeted metabolomics of bacterial siderophores: evidence of an unprecedented desferrioxamine pathway

**Fig. 4:**
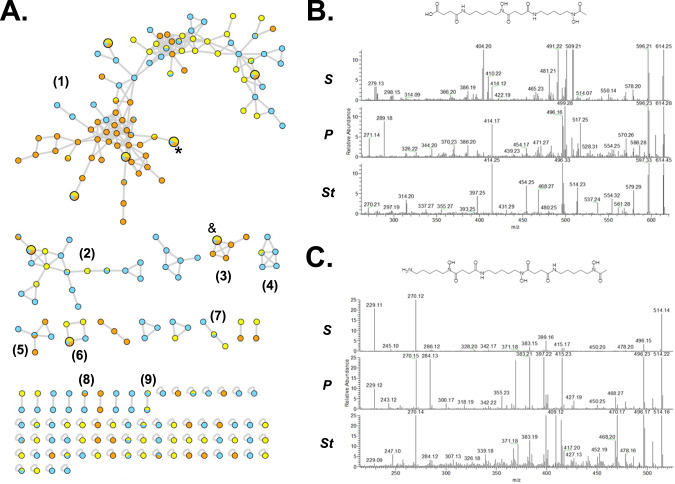
Conserved chemically divergent siderophores present in core taxa. **A** GNPS networks revealed 9 conserved clusters of metal-chelating metabolites produced by core bacteria of the genera *Pantoea* (Blue), *Serratia* (Yellow), and *Stenotrophomonas* (Orange). Four of these clusters included at least one metabolite shared amongst all three genera, but cluster 1 had four of these metabolites. Ferrioxamines H (*) and B (&) were identified in clusters 1 and 3, respectively, after a combined manual and GNPS annotation. Selected metabolites of each group, and their MS features, are provided in Table S13. **B** Ferrioxamine H and **C** Ferrioxamine B. MS/MS spectra of these known siderophores was manually analyzed and compared with standards and databases to confirm the validity of the GNPS annotation. *S* *Serratia*, *P* *Pantoea*, *St* *Stenotrophomonas*.

To explore the chemical diversity of siderophores in the isolated strains, Global Natural Products Social (GNPS) molecular networks [70] were constructed using MS data obtained from pooled peaks collected after HPLC separation (Fig. S12). To ensure that metal-chelating metabolites were targeted, we adopted a differential growth condition and analytical sample preparation with +/- ferric iron, as we have previously done in other microbial ecology studies [65]. Identification of metal chelating metabolites revealed 9 clusters of related compounds, with 7 of these metabolites conserved between the three bacterial core genera *Pantoea, Serratia*, and *Stenotrophomonas* (Fig. 4A, Table S13). Among these, we identified as conserved the siderophores ferrioxamine H ([M-2H+Fe]^+^ = 514.1717 *m/z*, cluster 1, Fig. 4B) and B ([M-2H+Fe]^+^= 614.2715 *m/z*, cluster 3, Fig. 4C), which are the succinylated dimer and the acetyl-capped trimer, respectively.

Unexpectedly, however, a putative desferrioxamine (*des*) BGC was not detected in any of the assembled metagenomes of the co-cultures, nor in the genomes of the isolated taxa that were sequenced independently. Our unsuccessful genome mining efforts included antiSMASH analyses; direct mapping of the reads of all six genome sequencing libraries against the *des* BGC from a close relative known to produce desferrioxamines, *Pantoea agglomerans* (MiBiG ID: BGC0001572); and identification of the Fur-dependent iron boxes implicated in iron metabolism regulation and previously exploited for siderophores genome mining [73]. The results of the latter analysis identified loci potentially involved in iron metabolism, of which some included biosynthetic enzymes and/or receptors, but were dissimilar to the DesABCD enzymes or the DesE receptor (Table S14). Together, these results provide evidence that diverse metal-chelating metabolites are present in the gut microbiome of cycad-feeding insects.

## Discussion

According to the 16 S community surveys, the core gut microbiomes of Coleoptera tended to be richer than those of Lepidoptera, which is consistent with previous reports that lepidopteran larvae typically do not host large resident bacterial communities [33, 74]. However, we found that a cross-species core microbiome is widespread among cycadivorous insects—including several lepidopterans—regardless of geographic location, insect order, life stage, host use, or feeding guild. Several of these core bacteria belong to clades that engage in beneficial and pathogenic associations with plants and insects, such as *Pantoea* and *Stenotrophomonas*. Although it is possible that similar microbiome convergence may be a more widespread feature of insect faunas that feed on chemically distinctive host plants, as postulated by the so-called “gut microbial facilitation hypothesis” [19], most insect microbiome surveys focus on a single species or group rather than on the microbiota derived from a community of herbivores, making it difficult to compare our results to those from similarly specialized insect faunas. For example, it would be interesting to compare our results with pine-feeding insects and their apparently convergent gut microbial associates that aid in the detoxification of terpenes [75–77] if complete and comparable data sets become available.

Co-culturing of microbial sub-communities, the key feature of the *EcoMining* approach, revealed that BMAA does not affect bacterial community composition. This is to be expected, as the insects’ gut lumens should contain BMAA from the insects’ food plants, and any bacteria that cannot survive in BMAA-rich environments are likely naturally excluded from these communities. Genome and metagenome mining revealed a rich biosynthetic potential, including a set of core BGCs found in all three insect species. Although only three insect species were included in the BGC analysis, all 12 insect species from the global 16 S survey contained the same bacterial OTUs from which the core BGCs were found. It may therefore be the case that the specific biosynthetic potential observed in the gut microbiomes of *Eumaeus*, *Rhopolotria*, and *Pharaxanotha* is widespread among cycadivorous insects, and could potentially contribute into the ability of these microorganisms to tolerate BMAA. Further BGC analysis predicted a high diversity of metal-chelating metabolites, e.g., siderophores, which are low molecular weight metabolites secreted by bacteria to chelate metals, often in response to iron deficiency, but which also play many ecologically relevant roles within bacterial communities [78]. As for the microbiomes of cycad-feeding insects, it remains to be seen if these siderophores are specific to core bacterial taxa or are common features of other bacteria within insects’ gut microbiomes.

Several catechol-type siderophores were predicted from the genomes of bacteria known to be enterobactin-like siderophore producers, such as *Pantoea* and *Stenotrophomonas* [79, 80]. Based on this evidence, the availability of iron (and potentially also other metals) may drive the diversification of these specialized metabolites within the insects’ gut microbiomes, as was recently shown in *Serratia plymuthica* with its combinatorial siderophore biosynthetic potential from polyamines [81]. Given that previous research into insect-siderophore interactions is limited, it is unclear whether the rich metal-chelating diversity observed within the microbiomes of cycadivorous is a widespread feature of insect gut microbiomes, or if it is limited to insects with specialized diets. Semi-untargeted metabolomics and molecular networking of metal-chelating metabolites produced by selected bacteria taxa showed signs of chemical diversification, consistent with the genetic diversity revealed by (meta)genome mining. Carotenoid-like aryl polyene biosynthetic systems, on the other hand, were found to be conserved and remain to be investigated, but it is interesting to note that they have been implicated in honeybee resistance to pathogenic fungi [82].

It is unknown how siderophores in the guts of cycadivorous insects might impact host fitness. A beneficial role of siderophores has been observed in plants, whereby siderophores produced by commensal or mutualistic bacteria can reduce growth of plant pathogens [83]. While research into siderophore-insect interactions is limited, previous studies have detected bacterial siderophores in the guts of mosquitoes, honey bees, grasshoppers, and moths [84–87]. In each of these cases, however, siderophore production was implicated in bacterial pathogenicity with clear costs for host fitness. Indeed, for many phyto- and entomopathogenic bacteria, siderophore production contributes to host colonization and microbial virulence, for example by competing with the host for iron and other essential micronutrients that are typically limited in plants. Without detailed assessments of the availability of iron and other metals to cycadivorous insects, it is unclear whether siderophore-producing bacteria may similarly compete with their hosts for iron. It also remains to be determined whether the siderophore chemical diversity measured here is different to that seen in taxonomically-related bacteria inhabiting different niches, a tantalizing challenge just starting to become accessible [88].

An interesting possibility is that siderophore-producing bacteria could provide protective benefits for cycadivorous insects by mediating chemical interactions between metal ions and BMAA inside the gut lumen. BMAA is also capable of chelating iron and other metals [89], and the formation of BMAA-metal ion complexes has been proposed as one possible cause of neurotoxicity in humans [90–92]. The chelation of divalent metal ions alters the equilibrium between BMAA and its corresponding carbamate adducts, which are required for binding to glutamate receptors and exerting neurotoxic effects [91]. Within the insect gut, the affinity of siderophores, such as desferrioxamines, for metal ions is likely to be higher than that of BMAA, such that bacterial siderophores may compete with BMAA for limited iron (and/or other metal ions serving as enzyme co-factors) within the insect gut lumen. In turn, this would alter the equilibrium between BMAA and its carbamate adducts affecting BMAA’s toxicity within the gut. This hypothesis warrants further investigation. A somewhat analogous scenario is found in the plant coumarins, e.g., the highly toxic and biologically active scopoletin, implicated in shaping the root microbiota by a ‘secondary’ siderophore activity [93, 94] that acts in concert with the bacterial redox-active phenazines produced by *Pseudomonas* [95].

Chemical diversification of bacterial specialized metabolites in the guts of cycadivorous insects is likely the result of complex ecological and evolutionary forces, including the biomolecular and physicochemical features of natural products synthesized by the insect and/or plant hosts, and may lead to novel and unexpected metabolic functions [96]. Within this context, iron-limitation is likely not the only factor driving siderophore production within the insects’ bacterial communities. Instead, we hypothesize that metal-chelating metabolites, e.g., siderophores, serve other functions besides chelating metals as a means for nutrient acquisition, as recently suggested in other systems [97]. That this genetic and chemical diversity is present in bacterial taxa found across all 12 of the insects we surveyed suggests that these specialized metabolites are a key feature of cycadivorous insects’ gut microbiomes. Future studies that take a plant-centered approach to surveying insect microbiomes, in combination with functional investigations, may ultimately yield more insight into the role of insects’ gut microbiomes in facilitating convergent adaptation to specific hosts.

### Supplementary information

Supplementary Information

Supplementary Tables S1–S14

## Data Availability

Raw sequence data and genome assemblies used in this study are deposited in the NCBI archive under accession number PRJNA961367. 16 S analysis code, biom file, phylogenetic tree, and metadata files are available on the Harvard Dataverse at 10.7910/DVN/OUSYHA
